# Therapeutic Adherence Promotion Program for Severe Mental Illness: The ADHERA Study Protocol

**DOI:** 10.3390/bs16030436

**Published:** 2026-03-17

**Authors:** José Luis Palomo-Ruiz, Carmen Artés, Santiago Ovejero, Enrique Baca-García, Alejandro Porras-Segovia

**Affiliations:** 1 Department of Psychiatry, Hospital Universitario General de Villalba, 28400 Collado Villalba, Spain; jlpalomoruiz@gmail.com; 2 Translational Psychiatry Research Group, Fundación Jiménez Díaz Health Research Institute, 28015 Madrid, Spain; carmenartesdelasheras@gmail.com; 3 Department of Psychiatry, Hospital Universitario Fundación Jiménez Díaz, 28040 Madrid, Spain; santiago.ovejero@quironsalud.es; 4 Department of Psychiatry, Universidad Autónoma de Madrid, 28049 Madrid, Spain; 5 Department of Psychiatry, Hospital Universitario Infanta Elena, 28342 Valdemoro, Spain; research.fjd@gmail.com; 6 Centro de Investigación Biomédica en Red de Salud Mental (CIBERSAM), Carlos III Institute of Health, 28029 Madrid, Spain; 7 Department of Psychiatry, Universidad Católica del Maule, Talca 3460000, Chile; 8 Department of Psychiatry, University Hospital of Nimes, 30900 Nimes, France; 9 Department of Psychiatry, University Hospital Rey Juan Carlos, 28933 Mostoles, Spain; 10 Department of Health Sciences and Neurosciences, University Carlos III, 28911 Madrid, Spain; 11 Research Unit U1191 (Environment, Biomarkers, Neuropsychatry, Institute of Functional Genomics), Institut National de la Santé et de la Recherche Médicale (Inserm), 34000 Montpellier, France

**Keywords:** schizophrenia, bipolar disorder, therapeutic adherence, electronical health records, medication

## Abstract

Treatment adherence can improve the prognosis of severe mental illnesses. Self-report questionnaires are the main tools to measure it. However, a new assessment method has emerged: the electronic prescription system. This enables us to verify whether patients have collected their medication from the pharmacy, providing an objective measure of adherence. The ADHERA study aims to: (1) Compare digital self-report questionnaires with the electronic prescription system. (2) Identify factors associated with non-adherence. (3) Evaluate the effectiveness of an adherence-promoting intervention. This intervention will consist of the identification of patients with poor adherence and a subsequent program of psychoeducation led by clinical psychologists. Adherence will be reassessed six months later to evaluate the intervention’s effectiveness. By identifying key sociodemographic and clinical factors associated with non-adherence, this project will inform targeted interventions to support patients with schizophrenia and bipolar disorder. Furthermore, the psychoeducational telehealth program may represent an effective, scalable, and patient-centered strategy to improve long-term treatment adherence and clinical outcomes. If successful, this model could be implemented in other regions and chronic conditions, contributing to a more efficient and patient-focused healthcare system.

## 1. Introduction

### 1.1. Relevance and Impact of the Problem

Schizophrenia and bipolar disorder (BD) are severe mental health conditions affecting up to 1% and 2.4% of the global population, respectively (Merikangas et al., 2011; Marder & Cannon, 2019). Both disorders are characterized by disabling symptoms, including psychosis and mood dysregulation, and significantly impair patient functionality, caregiver well-being, and productivity. This creates a substantial economic burden, largely driven by indirect costs such as lost productivity, unemployment, and caregiver strain. For instance, BD is one of the top 10 leading causes of disability worldwide (Ferrari et al., 2016) and costs an estimated $45 billion annually in the US (Hirschfeld & Vornik, 2005), while schizophrenia incurs an even higher cost of $155.7 billion, with unemployment and caregiving-related productivity loss as major contributors (Cloutier et al., 2016). These financial burdens are exacerbated by high rates of treatment nonadherence, with more than half of affected patients experiencing it (Gibson et al., 2013). This non-adherence often stems from medication side effects, cognitive impairment, stigma, or a lack of insight into the illness (Haddad et al., 2014).

### 1.2. Treatment Adherence in Severe Mental Illness

Antipsychotic medication is one of the primary therapeutic tools in managing schizophrenia and bipolar disorders. Both conditions are chronic central nervous system disorders that impose a significant social and healthcare burden. It has been well established that treatment adherence substantially improves the prognosis of these illnesses (Loots et al., 2021). While non-adherence is a challenge across all mental health conditions, it is particularly critical in schizophrenia and bipolar disorder, where discontinuation rates reach 40–60% (Valenstein et al., 2006; Semahegn et al., 2020). In these disorders, medication adherence is the primary predictor of relapse and suicide risk, making them the priority focus for this digital monitoring protocol. Moreover, the clinical consequences of non-adherence in SCZ and BD, such as high rates of emergency re-hospitalization and neuroprogression (Berk, 2009), represent a disproportionate burden on the healthcare system (Pennington & McCrone, 2017).

Beyond psychosocial determinants, the promotion of adherence must be understood within contemporary pathophysiological frameworks. Emerging models suggest that the clinical stability provided by continuous pharmacological treatment is essential for maintaining neurobiological homeostasis. Specifically, stable treatment may protect the glymphatic system, which clears metabolic waste in the brain. As highlighted by Barlattani et al. (2025), disruptions in these clearance mechanisms and inflammatory processes are increasingly linked to relapse vulnerability in psychiatric disorders. Therefore, improving adherence in the ADHERA study may potentially not only stabilize behavior but also preserve critical neurobiological clearance systems.

Until relatively recently, the main methods available for measuring treatment adherence were self-report questionnaires, which could be administered either on paper or electronically via platforms such as the Patient Portal. This digital communication platform, operational in some hospitals, allows patients to access appointments and reports and to respond to online questionnaires known as PROMs (patient-reported outcome measures), including medication adherence surveys. A major limitation of these self-report tools is their susceptibility to bias: patients with poor adherence may claim they are taking their treatment when they are not, to avoid intervention from healthcare staff. Also, these questionnaires provide only a cross-sectional picture, whereas continuous long-term monitoring would be more accurate and useful. With the advent of digital electronic prescriptions (Roden et al., 2012), a novel approach to assessing adherence has emerged in several countries, including Spain. In the Community of Madrid, the Single Prescription Module (Módulo Único de Prescripción, MUP) provides access to the patient’s pharmacological history and enables prescription management on this platform. Additionally, this prescription system is interoperable with those of other Autonomous Communities across the country. The MUP allows verification that patients have collected their prescribed medications from the pharmacy, providing an objective method to assess treatment adherence. A critical distinction must be made between self-report adherence measures and dispensing-based data. While self-reports are cost-effective and provide insight into the reasons for non-adherence, they are frequently confounded by recall bias and social desirability, leading to an overestimation of compliance (Nguyen et al., 2014). In contrast, new dispensing data processing tools, such as the PRE2DUP-R package (Tanskanen et al., 2015; Vattulainen et al., 2025), provide an objective, longitudinal record of treatment acquisition. Although dispensing data does not guarantee the actual ingestion of the medication, it serves as a more reliable predictor of clinical relapse than subjective scales in severe mental illness and has been validated both in serum concentration and comprehensive drug treatment interview (Forsman et al., 2018; Krousel-Wood et al., 2015; Taipale et al., 2016).

Recent studies have further expanded the landscape of digital adherence monitoring. Randomized trials have demonstrated that digital medication systems can significantly improve adherence in community patients with severe mental disorders (Zhou et al., 2025). Scoping reviews have identified a growing evidence base for app-based and eHealth interventions targeting antipsychotic adherence in schizophrenia (Wu et al., 2023), although an international expert consensus has highlighted the ongoing need for validated standards and robust efficacy data in this field (Smith et al., 2024).

### 1.3. Previous Literature and Rationale

Numerous studies have explored the concept of treatment adherence in schizophrenia (Acosta et al., 2012). For instance, the study by Kim et al. (2020) examined adherence and illness insight within the context of the CATIE antipsychotic treatment clinical trial. They found that impaired insight into the illness was a key predictor of earlier nonadherence to antipsychotic medication, underscoring the importance of interventions aimed at improving illness awareness to support long-term treatment adherence in schizophrenia.

Treatment adherence in BD has been widely studied; however, its critical determinants remain uncertain, as demographic and illness-related factors have consistently failed to fully account for or predict non-adherence (Chakrabarti, 2016). The latter suggested a patient-centered approach, highlighting the influence of attitudes toward medication, therapeutic relationships, stigma, and access to care—factors that may better explain non-adherence and guide more effective interventions. Additionally, psychological traits such as affective temperaments are increasingly recognized as relevant factors in adherence. Depressive temperaments have been associated with lower medication adherence, although self-care abilities may mitigate this association (Visalli et al., 2024), supporting the need for personalized strategies.

However, due to the relatively recent implementation and widespread adoption of electronic prescribing modules in many countries, fewer studies have utilized this method to assess treatment adherence.

### 1.4. Originality and Innovation

Due to the relatively recent implementation and widespread use of electronic prescription modules, there are very few comparable studies available, and none with our specific approach in the Spanish population. Therefore, our project offers significant innovation relative to the existing body of evidence.

Additionally, the project will incorporate digital elements in both the assessment of risk factors and the telematic intervention we will offer, leveraging new technologies to benefit patients.

Finally, the psychoeducational intervention will include a component of the “peer support” approach, in which stabilized patients contribute their own testimonies to help improve adherence in others. Such interventions have been shown to be effective in individuals with schizophrenia (Orsi et al., 2021) and bipolar disorder (Kelly et al., 2019), and may help ensure that the intervention is not perceived as coercive. The goal is not to “scold” patients for nonadherence but to help them understand the long-term benefits of sustained treatment adherence. The intervention is designed to respect patient autonomy and the right to refuse treatment. The program focuses on health literacy and shared decision-making, empowering participants to align their therapeutic choices with their personal recovery goals.

The ADHERA intervention is grounded in the Health Belief Model (HBM) and Motivational Interviewing principles. The conceptual model posits that the intervention influences adherence through a specific pathway: first, by improving clinical insight (knowledge of the disorder); second, by shifting attitudes (reducing perceived barriers and increasing perceived benefits of treatment); and third, by enhancing behavioral regulation (developing routines and digital self-monitoring). This progression is designed to transform adherence from a reactive behavior driven by external clinical pressure into a proactive health-seeking behavior.

### 1.5. Study Objectives

The objectives of our project are as follows:

1. To validate self-reported digital adherence questionnaires using dispensing data from the MUP on antipsychotic medication as the gold standard.
2. To identify factors associated with poor adherence by exploring correlations between various sociodemographic variables—available through the Patient Portal—and medication exposure as measured via the MUP.
3. To evaluate the effectiveness of the intervention by reassessing patient adherence six months after the intervention.

### 1.6. Working Hypotheses

Our working hypotheses are:

1. Pre-post comparison: Treatment adherence will increase following the intervention.
2. Risk factor association: Statistically significant risk factors associated with poor adherence will be identified.
3. Validity: The PROMs (Patient-Reported Outcome Measures) used will demonstrate concurrent validity with the objective data provided by the MUP.
4. Feasibility and acceptability: Both the intervention and the overall study will be well tolerated and positively received by participants.

## 2. Materials and Methods

### 2.1. Setting and Design

This project will be carried out in the Psychiatry Departments of four hospitals in the Community of Madrid associated with the Instituto de Investigación Sanitaria Fundación Jiménez Díaz: Hospital Universitario Fundación Jiménez Díaz, Hospital Rey Juan Carlos, Hospital General de Villalba, and Hospital Infanta Elena. This protocol combines three complementary methodological perspectives: (1) a validation study; (2) an observational study; and (3) a single-arm pre-post study to evaluate the intervention. All patients identified with poor adherence will receive the same psychoeducational program; the pre-intervention measurement will serve as a comparator.

**Figure 1:**
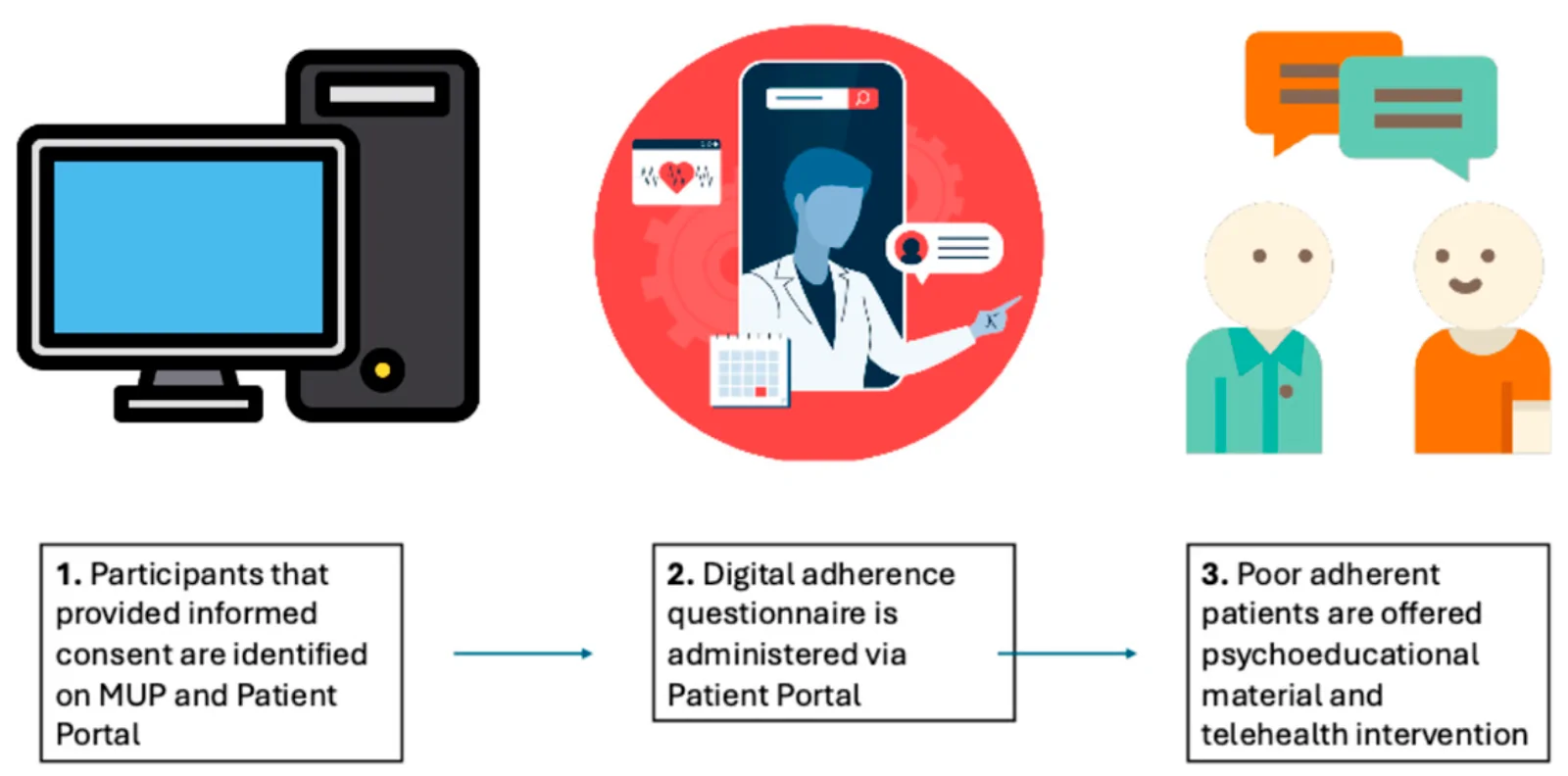
Outline of the ADHERA project.

Figure 1 shows the outline of the project.

This protocol describes a study with three design perspectives to achieve our objectives:

1. Validation study to explore the accuracy of digital self-report questionnaires.
2. Observational study for identifying factors associated with non-adherence.
3. Pre-post study to assess the effectiveness of the intervention

### 2.2. Sample

Our sample consists of patients with a diagnosis of schizophrenia and/or bipolar disorder.

Inclusion criteria:

- Diagnosis established using ICD-11 clinical criteria (World Health Organization).
- Aged 18 years or older.
- Registered on the Patient Portal.
- With an active prescription for antipsychotic drugs in the MUP.

To participate in the study, patients must have provided informed consent when registering through the Patient Portal, which allows access to their personal data for clinical and scientific purposes.

Exclusion criteria:

- Aged under 18 years old.
- Unable to provide consent for medical or legal reasons.
- Not registered on the Patient Portal.
- No active prescription for antipsychotic drugs in the MUP.
- Severe cognitive impairment, as determined by clinical judgment, that prevents the patient from providing informed consent or interacting with digital tools.

### 2.3. Sample Size Calculation

According to our preliminary data, there are 5458 patients in our hospitals diagnosed with schizophrenia or bipolar disorder and with active antipsychotic dispensing registered in the MUP. Of these, an estimated 90% are registered on the Patient Portal, yielding an initial estimated sample of 4900 people.

Considering that completion of self-administered questionnaires on the Patient Portal is estimated to be around 30%, we expect an approximate sample of 1640 patients to perform the validation calculations for the adherence scales.

The sample size was calculated using G*Power (v3.1) for a one-sample means test (alpha = 0.05, power = 95%, effect size d = 0.5), yielding a minimum of 52 participants. This was based on previous studies exploring the ability of educational interventions to improve medication adherence in the medium term (Loots et al., 2021). Accounting for an anticipated dropout rate of up to 50%, the adjusted minimum is 104. We therefore set a recruitment target of 110 participants, well within the estimated pool of 656 patients expected to present with poor adherence.

### 2.4. Procedure and Intervention

Project is expected to start in September 2026. We will access the MUP database and cross-reference it with data from the Patient Portal to identify our target sample, that is, those patients who received antipsychotic dispensations who also appear as active users of the Patient Portal. Within the entire sample, we will identify factors associated with adherence issues in the six months prior to the study. The entire sample will then be invited via a message through the Patient Portal to complete the self-report adherence questionnaires.

Finally, the subset of the sample identified with adherence problems will be the target sample for the intervention. Adherence problems to antipsychotics in people with schizophrenia or bipolar disorder are around 40–50%, although the variability in the studies is considerably high (Lingam & Scott, 2002). From the identified pool of patients with poor adherence, a recruitment target of 110 participants has been established. This sample size was determined using G*Power (v3.1) for a one-sample means test (alpha = 0.05, power = 95%, effect size d = 0.5), which requires a minimum of 52 participants (Loots et al., 2021). The final target of 110 accounts for an anticipated dropout rate of up to 50%, ensuring sufficient statistical power despite the high attrition typically observed in this clinical population.

**Figure 2:**
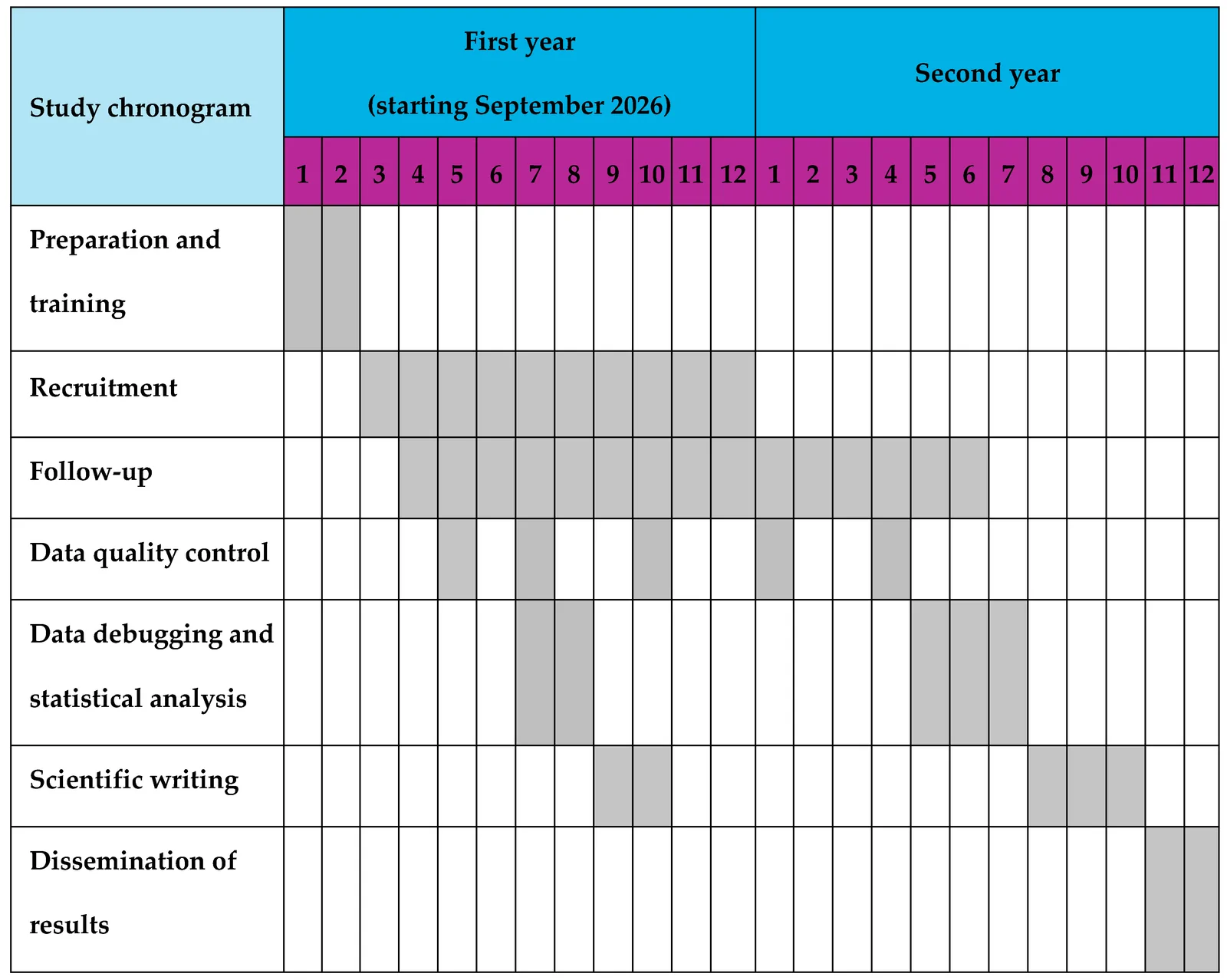
Timeline of the ADHERA project.

The intervention program will be designed by clinical psychologists with experience in treating patients with bipolar disorder and schizophrenia after a review of the best protocols available in the literature. Presumably, participants will receive psychoeducational materials on the importance of adherence and the potential for mishap management. Tablet handling tips and medication-taking schedules will be reviewed. Participants will also be invited to a telehealth intervention comprising 10 online group psychotherapy sessions delivered via videoconference. These sessions will include cognitive-behavioral measures to manage adherence problems, and adherence will be monitored. Psychotherapy sessions will be led by clinical psychologists, and two sessions will feature testimonials from stabilized patients (See Figure 2).

### 2.5. Outcomes

The study’s main outcome is treatment adherence, which was measured through the Morisky-Green 7-item scale (Morisky et al., 1986) and the Haynes-Sackett question (Sackett & Snow, 1979). Additionally, MUP will be used to calculate the percentage of time patients were exposed to antipsychotic treatment over the previous six months, measuring the effectiveness of the intervention. We will also use the MUP to record prescriptions for other medications, including those for general medical conditions.

This study will also gather sociodemographic and clinical data available through the Patient Portal, including sex, age, date of birth, urbanicity, and the presence of physical comorbidities.

### 2.6. Databases

Participant-identifying data (names and surnames, clinical record numbers) will be pseudo-anonymized using a code to prevent access to personal information. Only the minimum necessary data will be collected for study purposes, including sex, age, other sociodemographic factors, diagnosis, and scores obtained from clinical scales. To maintain data security, the head of the Psychiatry Department at Hospital Universitario Fundación Jiménez Díaz and a collaborating researcher will be the only individuals with access to individual subject data. This access will be facilitated via a tool that associates participant codes with clinical record numbers through an external table separate from the web-based participant database. The web-based participant database is hosted on Amazon Web Services, with all communications encrypted via HTTPS protocol. It is protected by the following security measures:

- Firewall protection provided by the hosting provider, allowing access only to known services.
- IPTABLES-based firewall as an operating system firewall.
- Linux server with SNORT intrusion detection system.
- Integrity control of various operating system files with AIDE.
- Multiple rootkit detection systems (CHKROOTKIT and RKHUNTER).
- External audits conducted with METAEXPLOIT and NESSUS.

During database or equipment maintenance operations, access to the remote maintenance console will only be permitted from allowed IP addresses (firewall-level filtering). A summarized log of all maintenance work performed (who/when/why/actions taken) will be maintained, as will a log of all accesses to maintenance consoles (log files).

### 2.7. Data Management

The study’s sponsor and participating center, responsible for processing personal data, will be the Psychiatry Department of Hospital Universitario Fundación Jiménez Díaz. Data will not be made publicly available to preserve patients’ privacy, but authors can be contacted for collaboration proposals.

The lawful basis for data processing is the patient’s explicit consent, as demonstrated by the Informed Consent Forms. Data will be retained for the duration of the study and for a maximum period of 20 years thereafter to fulfil any legal obligations arising from the relationship. For data processed for scientific research purposes, the Control Authorities of the Autonomous Communities may, upon request by the data controller and in accordance with established regulatory procedures, agree to the full retention of certain data, considering their historical, statistical, or scientific value in accordance with applicable legislation.

Access to personal information will be restricted to the study physician and their collaborators, health authorities, the Research Ethics Committee, and the sponsor’s monitors and auditors. These individuals are bound by the duty of confidentiality inherent to their profession when necessary to verify study data and procedures, always maintaining confidentiality in accordance with current legislation, including:

- Regulation (EU) 2016/679 of the European Parliament and of the Council of 27 April 2016 on Data Protection (GDPR).
- Organic Law 3/2018, of 5 December, on Personal Data Protection and Guarantee of Digital Rights.
- Provisions related to patient autonomy, information rights, and clinical documentation as outlined in Law 41/2002, of 14 November.
- Any other applicable and current regulations.

Therefore, participants’ identities will not be disclosed to any person, nor will data be communicated to third parties, except in cases of medical emergency or legal requirement. Prior to the commencement of this study, a risk prevention analysis was conducted by an independent entity (Grupo Ático34 S.L.).

### 2.8. Statistical Analysis

Statistical analyses will be performed using R software version 4.5.3 and SPSS software version 30.0, or the latest versions available. All tests will be two-tailed, with 95% confidence intervals, and the level of statistical significance will be set at a *p*-value less than 0.05. The Morisky and Haynes scales (Morisky et al., 1986) will be measured prior to the intervention and again at the end of the sessions, and changes will be analyzed using Pearson’s correlation and simple regression. On the other hand, we will compare the total duration of antipsychotic exposure in the 6 months prior to the intervention with that in the 6 months following the intervention. Similarly, we will compare the average doses to which the sample was exposed before and after the intervention by calculating the recommended daily doses received.

To validate self-report questionnaires, sensitivity, specificity, positive predictive value, and negative predictive value will be calculated using medication collection data from the MUP as the gold standard. ROC curves will be constructed to determine the empirically optimal cut-off for classifying adherence, using the Youden index and reported with 95% confidence intervals, with PRE2DUP-derived antipsychotic exposure as the reference standard.

To explore factors associated with higher or lower adherence, a logistic regression will be conducted, with adherence as the dependent variable. Adherence will be operationalized using the PRE2DUP method (Tanskanen et al., 2015; Taipale et al., 2016), generating two continuous outcomes: total duration of antipsychotic exposure (days) and average daily dose in DDDs (Tanskanen et al., 2015). These continuous variables will be used as dependent variables in all regression analyses. Furthermore, linear correlations will be performed between adherence levels (using percentage of adherence as a continuous variable) and various potentially implicated factors. To identify factors independently associated with non-adherence, a multivariable logistic regression model will be constructed, incorporating sociodemographic and clinical variables as independent predictors and adjusting for potential confounders (age, sex, diagnosis, comorbidities, and urbanicity). Simple logistic regression and Pearson correlations will be conducted as preliminary exploratory analyses to inform variable selection for the multivariable model. Pre-post changes in adherence outcomes—including PRE2DUP-derived exposure duration and average daily dose in DDDs, as well as self-report scale scores—will be analyzed using linear mixed-effects models, with time (pre- vs. post-intervention) as a fixed effect and subject as a random effect, adjusting for diagnosis, age, and sex.

## 3. Ethics and Dissemination

### 3.1. Feasibility

The expected duration of participation for each subject is approximately 6 months. The overall duration of the project is estimated at 2 years. The extraction and data analysis method has been previously explored to guarantee feasibility.

### 3.2. Applicable Regulations

This study has been approved by the Fundación Jiménez Díaz Ethics Committee and by Clinicaltrials.gov. The unique ID protocol is PIC 76-2013_FJD-HIE-HRJC approved on 28 January 2014 in act number 01/14.

This research will be conducted in accordance with the Declaration of Helsinki of the World Medical Association on ethical principles for medical research involving human subjects, as well as compliance with current legislation, including the European Parliament and Council Regulation (EU) 2016/679 of 27 April 2016 on the Protection of Personal Data (GDPR), Organic Law 3/2018 of 5 December on Personal Data Protection and Guarantee of Digital Rights, the provisions established in Law 41/2002 of 14 November, which regulates patient autonomy and rights and obligations concerning clinical information and documentation, and any other applicable laws and regulations.

### 3.3. Patient and Public Involvement

Patients and/or the public were not involved in the design, conduct, reporting, or dissemination plans of this research.

### 3.4. Personal Data Coding

Neither names nor any other identifiable personal information of participants will be published in any outputs derived from this research. Each participant will be assigned a unique code for identification purposes throughout the study, and no other personal data will be disclosed or used.

### 3.5. Cost and Compensation

There are no costs or financial compensations associated with participation in this study. All interviews and assessments will be conducted at no cost to participants. Participants will not receive direct clinical benefits or monetary compensation beyond the potential long-term benefits of contributing to a better understanding of the disease, which may lead to improved, more effective treatments in the future. While participation in the research protocol does not guarantee a direct clinical benefit, the psychoeducational intervention provided is designed to improve treatment adherence, which is positively correlated with a better long-term prognosis, including reduced relapse and hospitalization rates in both schizophrenia (Xia et al., 2011) and bipolar disorder (Colom et al., 2009).

### 3.6. Risks

The psychoeducational workshop presents no known side effects. Any discomfort experienced by participants during the study will be recorded.

### 3.7. Consent Procedures

Complete information about the study will be provided by a member of the research team prior to participant inclusion. Participants will be systematically given an information sheet, and the initial interview will aim to clarify any questions they may have. Parents or legal guardians will also be informed of the project details and will provide informed consent after reviewing the information sheet. It will be emphasized that participation or non-participation will not affect clinical care and that the study complies fully with privacy regulations. The investigator will ensure adherence to inclusion and exclusion criteria. The protocol does not specify a washout period.

## 4. Discussion

### 4.1. Expected Results

This study aims to identify the factors associated with poor treatment adherence by leveraging data available in electronic health records. Based on previous prospective studies in similar populations, we anticipate that approximately 40% of participants will show a meaningful improvement in adherence following the intervention (Misdrahi et al., 2023). This estimate is consistent with the moderate effect sizes (d = 0.5–0.7) reported for psychoeducational and motivational interventions in this population (Loots et al., 2021).

In addition to the direct benefits to participants, this project is expected to provide valuable insights into the factors influencing medication adherence in severe mental disorders and the reliability of self-reported adherence measures.

Ultimately, we hope our project will contribute meaningfully to improving the quality of life of individuals living with schizophrenia or bipolar disorder.

### 4.2. Dissemination Plans

This project is designed to be accessible to a large number of patients within our healthcare catchment area. Upon completion, findings will be disseminated through a multi-platform strategy:

- Scientific Publications: Between one and five peer-reviewed articles are expected to be published in open-access journals to ensure the broad availability of the results.
- Social Media Outreach: Our affiliated hospitals and collaborators maintain active profiles on Instagram, Facebook, X (formerly Twitter), and ResearchGate, which will be used to share study findings.
- Academic Conferences: Results will be presented at national and international psychiatry and psychopharmacology conferences through posters, oral presentations, and symposia.
- Media Coverage: Our institutional communications department will issue press releases aimed at making the study results accessible to the general public.

In terms of scalability, the methodology employed in this project is readily adaptable to other chronic conditions within our healthcare area, including both central nervous system and non-CNS disorders. Additionally, this model can be replicated in other healthcare regions with similar digital infrastructure, both within the Community of Madrid and across other autonomous communities. For instance, the Andalusian health system (Servicio Andaluz de Salud) employs the Diraya digital platform across all eight provinces in the region, and our team maintains active collaborations with hospitals throughout Andalusia. We also have ongoing partnerships with hospitals in Catalonia and the Principality of Asturias. Furthermore, we are open to establishing new collaborations with any institutions interested in implementing similar methodologies.

### 4.3. Limitations

Several limitations of the ADHERA study must be acknowledged. First, there is a potential selection bias inherent in the recruitment through the Patient Portal; participants who are already digitally literate or more engaged with the health system may be overrepresented, potentially limiting generalizability (Misdrahi et al., 2023). Second, our primary outcome relies on pharmacy dispensing data, which serves as a proxy for adherence but cannot confirm medication ingestion. While it remains a good proxy for objective adherence monitoring (Lam & Fresco, 2015), it cannot account for doses that are collected but not consumed. To address this issue, we will use the PRE2DUP-R R package (Tanskanen et al., 2015; Vattulainen et al., 2025). This method provides an objective approach to medication use and has been validated as a measure of medication use relative to both serum concentrations (Forsman et al., 2018) and a comprehensive drug treatment interview (Taipale et al., 2016). Third, telehealth interventions often experience attrition; although our peer-support component aims to mitigate this, some degree of dropout is expected. Finally, while the MUP system provides objective data, it does not capture medication provided through external samples or private clinical settings not integrated into the public electronic prescription network.

## 5. Conclusions

The ADHERA study will provide evidence on the reliability of digital self-report adherence questionnaires compared with objective dispensing data. By identifying sociodemographic and clinical factors associated with non-adherence, this project may inform targeted interventions for patients with schizophrenia and bipolar disorder. If the psychoeducational telehealth program proves effective, it could represent a scalable and patient-centered strategy to improve long-term treatment adherence, though this will depend on the empirical findings. Should results be favorable, the methodology may be adaptable to other regions and chronic conditions; however, formal replication studies would be needed before broader implementation could be recommended.

## Data Availability

The datasets used and/or analyzed during the current study are available from the corresponding author on reasonable request.
